# Supplementary material for: Resolving the full spectrum of human genome variation using Linked-Reads
Source: Genome Res. 2019 Apr;29(4):635–45. doi: 10.1101/gr.234443.118 (PMC6442396; doi:10.1101/gr.234443.118)
Supplement: Supplemental Material [file supp_gr.234443.118_Supplemental_longranger-2.2.zip › longranger-2.2.tar.bz2!/lr2.2-public/lib/stan/cmdstan/stan/doc/api/html/namespacemembers_func_o.html]

Stan: Namespace Members


|  |  |
| --- | --- |
|  | Stan  2.5.0  probability, sampling & optimization |


- Main Page
- Related Pages
- Namespaces
- Classes
- Files

- Namespace List
- Namespace Members

- All
- Functions
- Variables
- Typedefs
- Enumerations
- Enumerator

- a
- b
- c
- d
- e
- f
- g
- h
- i
- j
- l
- m
- n
- o
- p
- q
- r
- s
- t
- u
- v
- w


All Classes Namespaces Files Functions Variables Typedefs Enumerations Enumerator Friends Macros Pages

### - o -

- operator!()
  : stan::agrad
- operator!=()
  : stan::agrad
- operator\*()
  : stan::agrad
- operator+()
  : stan::agrad
- operator++()
  : stan::agrad
- operator-()
  : stan::agrad
- operator--()
  : stan::agrad
- operator/()
  : stan::agrad
- operator<()
  : stan::agrad
- operator<<()
  : stan::gm
- operator<=()
  : stan::agrad
- operator==()
  : stan::agrad
- operator>()
  : stan::agrad
- operator>=()
  : stan::agrad
- ordered\_constrain()
  : stan::prob
- ordered\_free()
  : stan::prob
- ordered\_logistic\_log()
  : stan::prob
- ordered\_logistic\_rng()
  : stan::prob
- owens\_t()
  : stan::agrad
  , stan::math


---

[ Stan Home Page ]

*© 2011–2014,
Stan Development Team.*
